# Clustering of Socioeconomic Data in Hong Kong for Planning Better Community Health Protection

**DOI:** 10.3390/ijerph182312617

**Published:** 2021-11-30

**Authors:** Zhe Huang, Emily Ying Yang Chan, Chi Shing Wong, Benny Chung Ying Zee

**Affiliations:** 1 Collaborating Centre for Oxford University and CUHK for Disaster and Medical Humanitarian Response (CCOUC), The Chinese University of Hong Kong, Hong Kong SAR, China; huangzhe@cuhk.edu.hk (Z.H.); cswong@cuhk.edu.hk (C.S.W.); 2 GX Foundation, Hong Kong SAR, China; 3 Centre for Clinical Research and Biostatistics (CCRB), The Chinese University of Hong Kong, Hong Kong SAR, China; bzee@cuhk.edu.hk; 4 Office of Research and Knowledge Transfer Services (ORKTS), The Chinese University of Hong Kong, Hong Kong SAR, China

**Keywords:** socioeconomic vulnerability, Health-EDRM, Hong Kong, cluster analysis, mortality

## Abstract

The concept of socioeconomic vulnerability has made a substantial contribution to the understanding and conceptualization of health risk. To assess the spatial distribution of multi-dimensional socioeconomic vulnerability in an urban context, a vulnerability assessment scheme was proposed to guide decision-making in disaster resilience and sustainable urban development to reduce health risk. A two-stage approach was applied in Hong Kong to identify subgroups among Tertiary Planning Units (TPU) (i.e., the local geographic areas) with similar characteristics. In stage 1, principal components analysis was used for dimension reduction and to de-noise the socioeconomic data for each TPU based on the variables selected, while in stage 2, Gaussian mixture modeling was used to partition all the TPUs into different subgroups based on the results of stage 1. This study summarized socioeconomic-vulnerability-related data into five principal components, including indigenous degree, family resilience, individual productivity, populous grassroots, and young-age. According to these five principal components, all TPUs were clustered into five subgroups/clusters. Socioeconomic vulnerability is a concept that could be used to help identify areas susceptible to health risk, and even identify susceptible groups in affluent areas. More attention should be paid to areas with high populous grassroots scores and low young-age score since they were associated with a higher mortality rate.

## 1. Introduction

Risk is a function of hazard, exposure, and vulnerability. As highlighted in the World Health Organization health emergency disaster risk management (Health-EDRM) framework [1], the understanding and managing of health risk in non-emergency times are vital to protecting health and safeguarding development. To fully comprehend health risk, not only the physical impact of a hazard that might affect people should be known, but also how diverse the impact might be on different social groups, which is determined by social systems and power. In contrast with exposure, which is usually associated with a geographical location, vulnerability is a state of well-being and is socially differentiated [2]. When faced with the same hazard, people in different social groups will vary in vulnerability. If a hazard has an absolute impact, vulnerability affects how the impact is relatively received. For example, compared to the middle class or the rich, the poor are more vulnerable to hazards.

Vulnerable groups are those who suffer more from the immediate impact of an extreme event, and those who are less resilient to an extreme event in terms of difficulty in reconstructing their livelihoods and health after the event. In general, vulnerability is closely correlated with socioeconomic status (e.g., income and housing) and social characteristics (e.g., age, gender, ethnicity, and extent of social networks) [3]. It might not be very meaningful to distinguish between socioeconomic status and social characteristics since they often overlap with each other. However, the concatenation of these two is essential for evaluating peoples’ combined vulnerability correctly since almost everyone has some capacities to make himself less vulnerable to hazards. For example, an old man with low education might appear vulnerable but living in a rich family could alter his vulnerable situation. Since policy and the re-distribution of resources can affect vulnerability, vulnerability in the economic and institutional context could be changed more easily at a temporal scale than exposure and physical hazard. Hence, consideration of social and economic vulnerability could be essential for studying human adaptation to disaster and health risks. There is a need to understand how social and economic vulnerability is associated with disaster and health risks, and such enhanced understanding can be used to inform policy makers to ameliorate such vulnerability and related health risks. Although vulnerability depends on context, and it might be overly simplistic to assume that vulnerabilities apply to all hazards, generic vulnerability, as opposed to hazard-specific vulnerability, could still be useful as a basis to indicate how well an area is equipped to cope with a variety of hazards and accompanied health risks [4].

A complex set of indicators determine generic vulnerability in different social contexts. According to Adger [2], the major contributors of vulnerability are poverty, resource dependency, inequality, and institutional adaptation. Since this framework was developed for national-level comparison, institutional adaptation might not be useful when resources are allocated by a single central government.

Firstly, the lack of access to resources is often the cause of vulnerability [5] and given that access to resources is difficult to measure, poverty provides a useful proxy for lack of access to resources. Income is a good proxy for poverty and is also directly linked to coping capacity in an extreme event. In addition, studies have reported more exposure of poor people to environmental hazards [6,7], though the causal relationship is not clear.

Secondly, resource dependency can be described as the reliance on local systems, lack of income source diversity, livelihood instability, and low resilience [2]. Working outside one’s home area is a sign of being independent of the local context and associated with enhanced livelihood stability. Further, both marital status and household size have been regarded as important in the division of household labor and family support [8,9,10]. With the steady increase in labor force participation of women, large household size and a high proportion of females in a household can diversify income sources. In addition, since women are more likely to adopt the role of a family caregiver across different cultures [11,12], a high female to male ratio is an important indicator of livelihood stability.

Thirdly, inequality affects vulnerability by channeling service provision toward those with accumulated assets and constraining communal allocation of resources. Unemployment, low car ownership, lack of housing tenure, and poor housing are all associated with deprivation [13]. Although public housing has been regarded as a tool for altering the long-term housing inequality in urban neighborhoods [14,15], it also reshapes the surrounding neighborhood and creates neighborhood income inequality by introducing a low-income group because public housing units are usually developed for the most socially deprived [16].

The indicators mentioned above mainly concern socioeconomic status; additional social characteristics are now quite standard in vulnerability research [3,4,17,18,19,20], including variables relating to demography, literacy and education, occupation, migration, and language. These social characteristics are related to the response to and recovery from disaster and contribute to constructing the concept of vulnerability to hazard. Although there is no consistent set of indicators for vulnerability assessment, age, gender, ethnicity, and socioeconomic status are generally included [18]. However, characteristics focusing on people with special needs or recovering from disaster might be neglected due to data availability and accessibility.

There are two types of vulnerability definitions referred to in the relevant literature: biophysical vulnerability and inherent vulnerability [21]. The former refers to the amount of (potential) damage to a community caused by a hazard, focusing on the ultimate outcome resulting from a hazard. The latter refers to a state or an inherent property of a community, which is independent of the hazard, focusing on the status before the hazard event occurs. The former is often measured by human casualty or monetary cost, while the latter is determined by poverty and housing quality. The second definition is used in this study. From the perspective of inherent vulnerability, a health outcome is a product of the interaction of hazard and vulnerability, rather than an element of vulnerability.

### The Case of Hong Kong

Hong Kong is a densely populated city with a population of more than seven million people and large income disparity. Given the close relationship between vulnerability, socioeconomic status and health, socially vulnerable areas are worthy of being identified and categorized for increased community health protection and resource allocation. To date, there has been limited research focusing on the comparison of socioeconomic vulnerability across locations in Hong Kong [22], and there is a lack of a robust and consistent set of indicators for assessing social vulnerability that facilitates comparisons among diverse locations. As the living environment plays an important role in one’s well-being, the housing problem is one of the most pressing issues that Hong Kong faces since Hong Kong’s housing is amongst the least affordable in the world; the small floor area housing dubbed “nano flats”, for example, is notorious [23]. Housing is, therefore, analysed as an indicator of inequality in this study.

As mentioned above, vulnerability-related data are multidimensional and correlated with each other. In view of the large number of variables, identifying a smaller subset that exhibit the strongest effects is easier for interpretation than presenting a substantial number of variables, which can be achieved by certain dimension reduction methods. In addition, identifying subgroups across areas with similar features using clustering methods, can often allow a better understanding of different areas. Subsequently, the government might choose appropriate measures or policies to implement in a particular area based on experience in other areas with similar patterns of features.

The objectives of this study are (1) to summarize, reduce and simplify a substantial number of socioeconomic dimensions into a few variables in Hong Kong (i.e., drivers of vulnerability), and (2) to spatially categorize all areas in Hong Kong into a small number of subgroups based on the variables generated in objective 1 to assess the spatial distribution of socioeconomic vulnerability in this urban city.

## 2. Materials and Methods

### 2.1. Data Scoping

**Figure 1:**
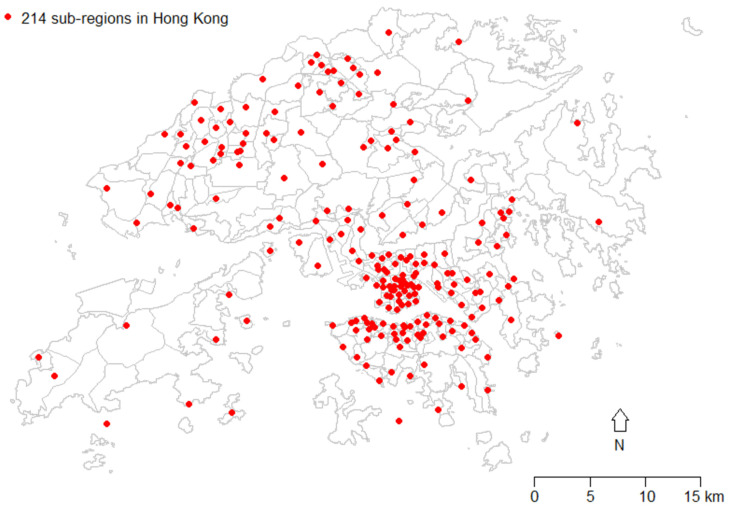
The map of 214 sub-regions in Hong Kong. Note: The red dots indicate the centroids of sub-regions, which are the geometric centers of the areas. Since some sub-regions contain multiple islands, the centers might be in the sea. In total, there were 214 red dots.

In Hong Kong, the Tertiary Planning Unit (TPU), demarcated by the Planning Department of the Hong Kong Special Administrative Region (SAR) government, is an appropriate geographical unit for the selection of neighborhoods for analysis/grouping, as it is linked with census data including various census-level variables on mortality, socioeconomic status, and social characteristics. In 2016, the whole land area of Hong Kong was divided into 291 TPUs (Figure 1). To protect data privacy, TPUs with less than 1000 persons were merged with adjacent TPUs when government statistics were released. Hence, the 291 TPUs were grouped into 214 sub-regions.

To sort areas with similar features in Hong Kong into a small number of clusters, socioeconomic data were collected for all TPUs/sub-regions, the unit of our analysis, from the 2016 population by-census from the Census and Statistics Department of the Hong Kong SAR government [24]. The socioeconomic data selected included demographic, educational, economic, household, and housing variables, characterizing different dimensions of the respective area. A total of 27 raw and computed variables were selected, which are commonly used in vulnerability-related research, and summarised with the relevant research that identified them (Table 1).

### 2.2. Statistical Analysis

A two-stage approach, performing clustering on the first few principal component score vectors [27], was used to identify subgroups among all the TPUs with similar characteristics. In stage 1, principal components analysis (PCA), one of the simplest and most robust ways of performing dimension reduction, was used for de-noising the socioeconomic data for each TPU based on the variables selected, which enables an easier understanding of the overall picture of the social and economic vulnerabilities, while in stage 2, a Gaussian mixture model (GMM) was used to partition all the TPUs into different subgroups based on the results of stage 1.

Stage 1: First, PCA was performed on the socioeconomic data after scaling the variables to have mean zero and standard deviation one, since the socioeconomic data to be used were on very different scales. So, the principal components were extracted from the correlation rather than the covariance matrix. The number of principal components extracted should contribute cumulatively to the explanation of the overall variance by more than 60% [28]. Principal components whose eigenvalues were less than 1 were excluded since they contributed less variance than the average [29]. A scree plot was produced; the “elbow” in the plot was used to help determine the number of principal components, i.e., where the slope in the scree plot changed from “steep” to “shallow”, indicating that increasing the number of principal components could not effectively contribute further to the overall variance. A Varimax rotation [30] was conducted to obtain a small number of large loadings and as many near-zero loadings as possible for better interpretation of the principal components.

Stage 2: To determine if there was sufficient clustering tendency to proceed with cluster analysis, the Hopkins statistic [31] was calculated before the subsequent analysis. If the Hopkins statistic is approximately 0.5, there is no meaningful clustering of the data; whereas if the Hopkins statistic is approximately 1.0, which means the first few principal component score vectors were amenable to clustering in this study, then all the TPUs would be partitioned into different groups through GMM. The Bayesian information criterion (BIC) was used to select the optimal number of clusters [29,32].

In addition, we calculated the Mahalanobis distance for each TPU from its cluster center, where the Mahalanobis distance measures how distant a point is from the mean of a multivariate Gaussian distribution, in contrast to Euclidean distance, by taking the covariance matrix of the data into consideration. As the square of this distance is distributed as a χ^2^ statistic, with degrees of freedom equal to the number of independent variables [33], which was the number of the principal components in this study, any TPU with a Mahalanobis squared distance larger than 3, the critical value using an alpha of 0.01, was considered as an outlier.

Although the socioeconomic data collected in each TPU were for town planning purposes rather than health monitoring, previous literature has suggested that TPU-level socioeconomic status affects health [22,34]. In this study, multiple regression analyses were used to attempt to link health data to the principal components derived, in order to investigate whether TPU is an appropriate unit for the selection of neighborhoods in further analyses. Regressing health data on principal components orthogonal to each other is also useful to avoid multicollinearity encountered when using the original socioeconomic data. R version 3.6.1 (R Foundation for Statistical Computing, Vienna, Austria) was used for all the analyses.

## 3. Results

TPUs 941, 942, and 943, located in the southwest corner of Hong Kong, were excluded in this study since the numbers of domestic households in each were too small and some related statistics derived based on such a small number of domestic households are not released from the government. Moreover, these areas were in a mountainous setting and considerably different from the others. Since these three TPUs were in the same sub-region, there remained 213 sub-regions, covering 288 TPUs, that were included in the subsequent analyses.

### 3.1. Principal Components

**Figure 2:**
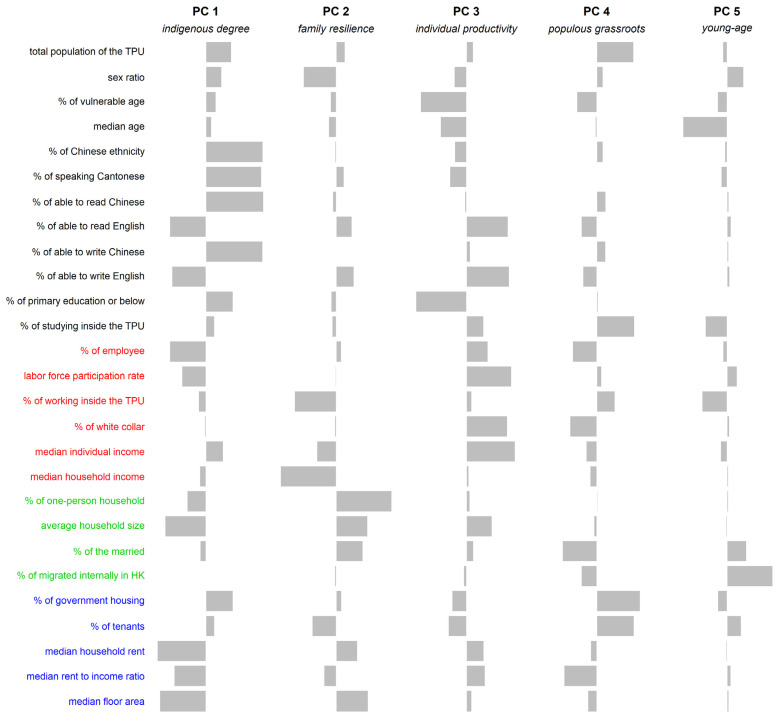
The loadings for the five principal components. Note: The bars depict each correlation coefficient between the original variable and principal component. The signs of the coefficients of the socioeconomic variables do not affect their importance in the principal component while the magnitude of them does. A longer bar indicates higher absolute correlation coefficient.

**Figure A1:**
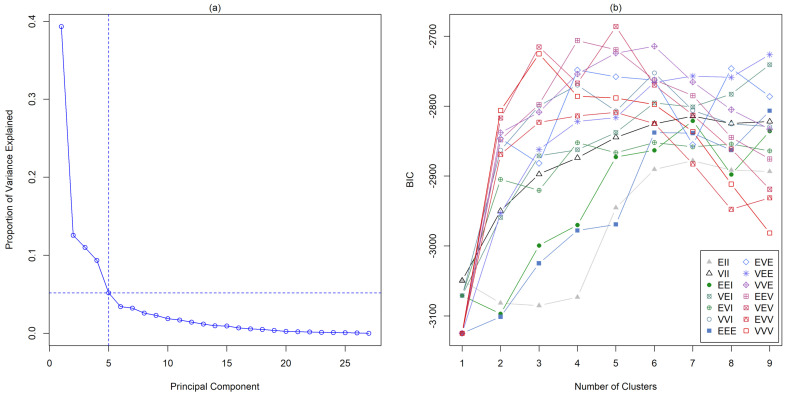
(**a**) Scree plot and (**b**) Bayesian information criterion (BIC) plot for Gaussian mixture model (GMM). Note: BIC defined in some literature about GMM is the negative of that defined in standard references, where smaller value of BIC is preferred. So, for GMM, a larger BIC value indicates a better model.

The results of the scree plot (Appendix A Figure A1a) for the sufficiency of the number of principal components suggested that a five-component solution was adequate to account for the observed covariance in the data among the 213 sub-regions in Hong Kong. In addition, all these first five components had variances (eigenvalues) greater than one and together explained about 77.4% of the total variance of the original 27 variables included, further supporting that the five-component solution was sufficient. The principal component loading matrix after the orthogonal rotation is shown in Figure 2.

Since principal component 1 (PC1) was dominated by the proportion of Chinese ethnicity, usual spoken language, and Chinese proficiency, while moderately affected by median household rent, median floor area of accommodation, household income, the proportion of employee, and English proficiency, this component was labeled, “indigenous degree”.

Principal component 2 (PC2) had its highest loadings on household size and moderate loadings on working outside the sub-region, gender ratio, median floor area of accommodation, and household income, and was labeled, “family resilience”.

The third principal component (PC3) was highly correlated with educational attainment and moderately with the proportion of white collar, the proportion of labor force, labor force participation rate, English proficiency, and individual income, so it was labeled, “individual productivity”.

Principal component 4 (PC4) was moderately related to the proportion of government housing, studying inside the sub-region, the proportion of tenants, population of the sub-region, the proportion married, and median rent to income ratio. We named it “populous grassroots”.

Principal component 5 (PC5) was mainly associated with the median age of the sub-region and the proportion of people having internally migrated. We named the last component “young-age”.

### 3.2. Principal Component Scores of TPUs

**Figure 3:**
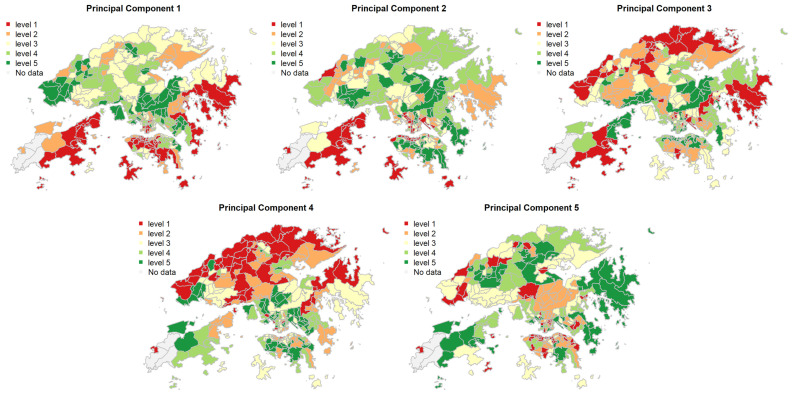
Principal component scores for the five principal components. Note: The five principal components were indigenous degree, family resilience, individual productivity, populous grassroots and young-age respectively. All principal component scores of the TPUs (Tertiary Planning Units) were categorized into five levels using the quantile classification method for data presentation. A five-level scale was selected, as it offers good balance of level differentiation and understandability, and highlights TPUs are in the top and bottom 20%. Higher level indicates higher principal component score.

The estimated scores of PC1 to PC5 for each sub-region were calculated and categorized into five levels, as shown in Figure 3. For example, considering PC1, which reflects the indigenous degree, the eastern and southern parts of Hong Kong were the least indigenous areas, whilst the western and central parts of Hong Kong were shown to be the most indigenous among all the studied TPUs. The fourth principal component score was the highest in northern Hong Kong because there were less government housing, fewer people studying inside the sub-region, and a lower proportion of tenants, while southern and central Hong Kong had more government housing, more people studying inside the sub-region, and a higher proportion of tenants.

### 3.3. Clustering for TPUs

**Figure 4:**
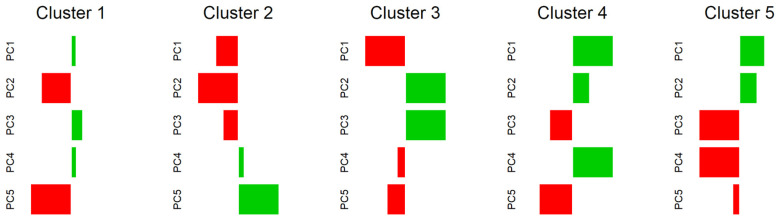
Mean scores for all the principal components of the respective clusters. Note: Green bars and red bars indicate positive and negative mean scores respectively. Longer bar indicates a higher absolute mean score.

**Figure 5:**
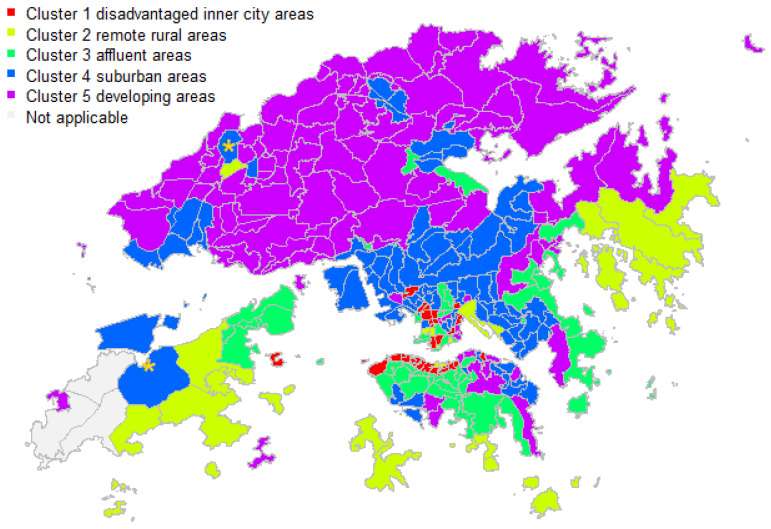
The five-cluster solution. Note: Two outliers were identified using gold asterisks, with Mahalanobis squared distance of 3.89 and 4.00, exceeding the critical value of 3. Both of these were in Cluster 4. Areas in grey located in southwest corner were the sub-region being excluded in the analysis.

The Hopkins statistic was approximately 0.83, which suggested that the data were arranged in tight clusters. GMM was then performed to group all the TPUs into a small number of clusters. The BIC indicated that a five-cluster model with covariances having different volumes and orientations but the same shape (i.e., VEV) provided the optimal solution. Plots of the BIC traces for models with different parameterizations of the covariance matrix are shown in Appendix A Figure A1b. In addition, the mean scores for the principal components of the respective cluster are illustrated graphically after min-max scaling in Figure 4. The five-cluster solution is shown in Figure 5.

Cluster 1 consisted of areas with the lowest young-age score and relatively low scores for family resilience. This cluster included the northern coast of Hong Kong Island, which embraces Sheung Wan and Wanchai, as well as Sham Shui Po, Mong Kok, and Tsim Sha Tsui in Kowloon.

Areas in Cluster 2 had the lowest principal component score in family resilience but the highest for young-age. This cluster contained remote areas such as Sai Kung and Hei Ling Chou.

Areas in Cluster 3 had the highest scores in family resilience and individual productivity components, and the least score in indigenous degree, including Repulse Bay, Deep Water Bay, and Kowloon Tsai.

Cluster 4 had the highest scores in populous grassroots and indigenous degree, with relatively low scores in individual productivity and the young-age components. It appeared that these areas were mainly located in the middle of Hong Kong and scattered in the north.

Cluster 5 included areas with the lowest populous grassroots and individual productivity scores, with relatively high indigenous degree scores. It appears that they were mainly located in the northern part of Hong Kong.

### 3.4. Using Principal Components to Predict Mortality Rate

**Figure A2:**
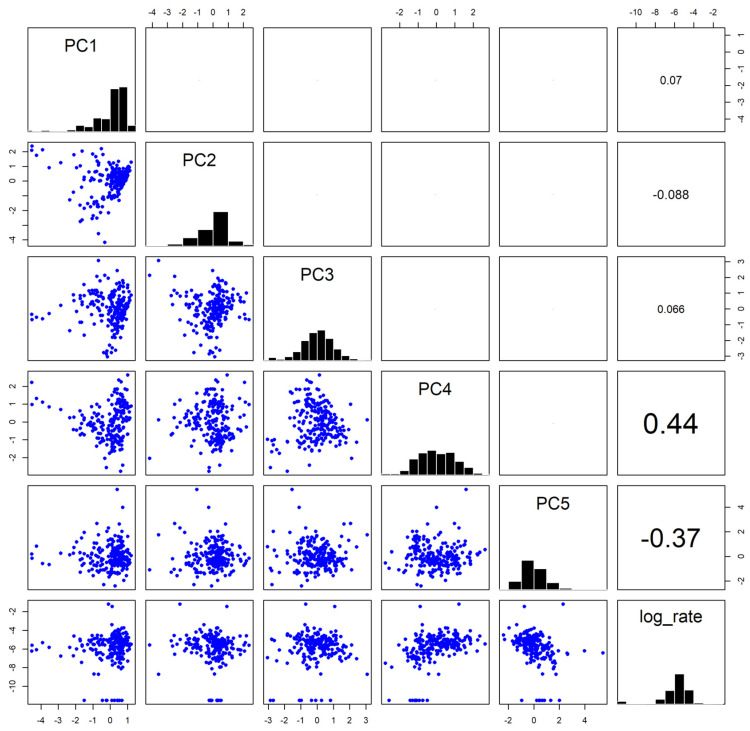
Relationships between principal component scores and log-mortality-rate. Note: The diagonal panels show the histograms of the principal components scores and log-mortality-rate; The lower panels show the scatterplots of the corresponding variables; The upper panels show correlation coefficients where the font size is proportional to the magnitude of the correlation coefficient.

**Figure A3:**
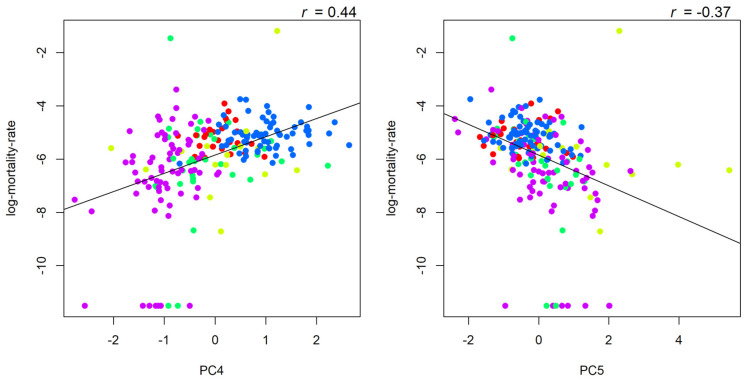

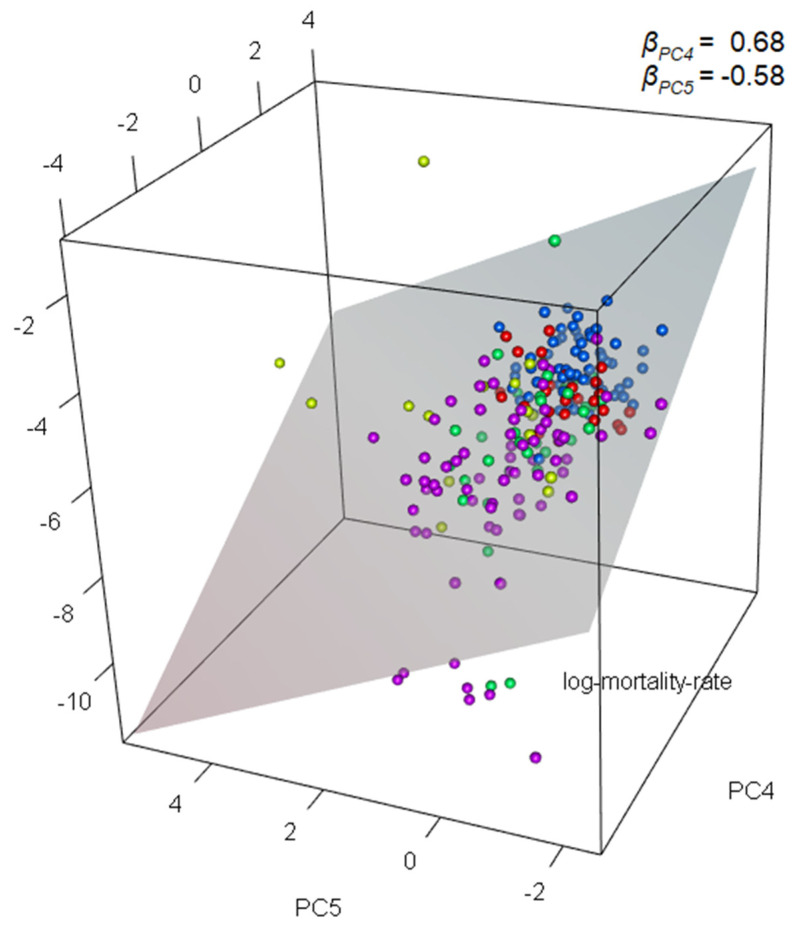
Regressing log-mortality-rate on PC 4 and PC 5 in simple linear models and multiple linear model.

To preliminarily test the reliability and usefulness of the principal components, we examined the log-mortality-rate by different sub-regions in 2016. We initially conducted a simple correlation analysis between the log-mortality-rate and the five principal component scores (Appendix A Figure A2). Components with small variance (i.e., the fourth and fifth principal components) had large correlations with the log-mortality-rate. There was a positive relationship between the log-mortality-rate and the populous grassroots principal component (*r* = 0.44, *p* < 0.001), but a negative relationship between log-mortality-rate and the young-age principal component (*r* = −0.37, *p* < 0.001). When we put these two principal components into a multiple linear regression, the model had an adjusted *R^2^* of 32.2%, and the coefficients of both principal components were highly statistically significant, with *β_PC4_* = 0.68 (*p* < 0.001) and *β_PC5_* = −0.58 (*p* < 0.001) (Appendix A Figure A3).

## 4. Discussion

This paper presents a two-stage approach, applying a Gaussian mixture model based on identified underlying dimensions of social vulnerability to demonstrate similarities and identify homogeneous subgroups among the TPUs in Hong Kong.

The concepts of socioeconomic vulnerability were adapted from Adger’s conceptual model [2], which was first developed to better understand adaptation to climate change impacts. The underlying dimensions of socioeconomic vulnerability included indigenous degree, family resilience, individual productivity, populous grassroots and young-age. The terminology for these five dimensions will require further development, but this is beyond the scope of this study. According to these five dimensions, all TPUs were clustered into five subgroups/clusters, namely: (1) disadvantaged inner city areas, (2) remote rural areas, (3) affluent areas, (4) suburban areas and (5) developing areas. This two-stage approach is applicable to different topics and is particularly useful when theoretical frameworks are not consistent. The results of this approach in this study capture specific features of the local context, providing insight into the observed situation with potential for translation into policy in Hong Kong.

### 4.1. Five Underlying Dimensions of Vulnerability

#### 4.1.1. Principal Component 1: Indigenous Degree

PC1 is mainly concerned with cultural capital vulnerability, which is about the access to international information and elitist circles. In Hong Kong, the Chinese community comprises the overwhelming majority, and Cantonese is the most widely used spoken language, especially among the Chinese community. However, like some countries in former British colonies, English is the principal language of many domains [35], and even a symbol of elitism among Chinese professionals [36]. For example, Hong Kong tertiary students, and even some secondary school students, were taught in English instead of Cantonese as a medium of instruction [37]. Hence, Cantonese is used for daily spoken communication, while English is the preferred official language for professional and business sectors [36]. In addition, the use of English in business and the professions has been regarded as one of the keys to Hong Kong’s economic success [38]. In Hong Kong, English language proficiency is an important factor in securing employment [39], and professionals occupying senior ranks were found to have a higher proficiency of English [35].

In the current study, TPUs with a high indigenous degree score were associated with low English proficiency, low household income, and small living area, but there is considerable heterogeneity among indigenous TPUs in Hong Kong in terms of other social and historical factors. Although there is little question that improving English proficiency increases local competitiveness in business and the professions, it may cause elitism, estrange communities, and compromise the effectiveness of public health measures among the Cantonese-speaking majority.

#### 4.1.2. Principal Component 2: Family Resilience

PC2 might be regarded as isolation vulnerability. In Chinese tradition, family is viewed as a resource that provides support and security to its members. The second component of vulnerability, (lack of) family resilience, was mostly related to household size, as well as household income and living area. The TPUs with the least family resilience score were mainly located on both shores of Victoria Harbor. In Hong Kong, the average household size was 2.8, and the proportion of one-person households was 18.3%. However, the proportion of one-person households in Wan Chai North was around 60%, while the average household size was less than two. In Central district, the proportion was 43.8% and a similar pattern was found on the northern coast of Hong Kong Island.

Given the increasing trend of living alone in Hong Kong (15.6% in 2001; 16.5% in 2006; and 17.1% in 2011) [40], this phenomenon becomes of public health interest since living arrangement affects the amount of physical and mental support received from family members. While family involvement plays an important role in early detection of disease and decision to seek help [41], previous studies related to living alone focused mostly on older people although there could be a large growth of living alone among younger populations [42] as a lifestyle choice.

#### 4.1.3. Principal Component 3: Individual Productivity

PC3 mainly focuses on income vulnerability. Individual productivity was related to individual’s education, occupation, and individual income. It may be surprising that the individual productivity scores were relatively low in the southern parts of Hong Kong Island (e.g., Repulse Bay and Deep Water Bay) since they were rich neighborhoods in Hong Kong traditionally. However, more than 20% of the population in these two areas were Filipino domestic helpers. If domestic helpers from other countries (e.g., Indonesia) were included, the proportion would increase to 30%. Due to the high proportion of foreign domestic helpers with relatively low individual income, the individual productivity scores were lowered in these areas. Hence, although household income was high in these areas, which is partially reflected in the high family resilience score, individual variations could be substantial.

In 2016, there were more than 350,000 foreign domestic helpers in Hong Kong, mostly females, with 54% from the Philippines, and 44% from Indonesia [43]. In addition, the Indonesian share of paid domestic helper markets in the eastern parts of Hong Kong Island was higher than that of the Filipino. Although foreign domestic helpers played an important role in helping Hong Kong families with household chores, their minimum wage was much lower than that of local domestic workers [44].

#### 4.1.4. Principal Component 4: Populous Grassroots

PC4 is essentially home ownership vulnerability. The component of populous grassroots was positively correlated with the proportion of government housing and proportion of tenants but negatively correlated with individual income. The unaffordable home ownership situation in Hong Kong is unlikely to change in the near future, and the government is relocating the grassroots population from the crowded urban areas to more spacious areas through the construction of government housing and related infrastructure in these suburbs so that people can afford the housing in terms of a low rent-to-income ratio. However, too many people in disadvantaged socioeconomic status living together creates a new vulnerability, as indicated by the significant correlation between the populous grassroots score and mortality, which was consistent with the findings of Lawder et al. [45] and Kandt et al. [46] about worse health outcomes in neighbourhoods with a larger percentage of public housing. In addition, tenants were also more likely to report poor self-rated health or anxiety [47]. But Kandt et al. also argued that the adverse health outcomes were contributed by the deprivation itself, and living in public housing was even an advantage when compared with deprived peers living in low-end, private rental housing (i.e., subdivided and cage dwellings). Detailed analyses were needed to separate the effect of public housing. On average, private housing tenants paid much higher rent (41% of household income) than households living in public housing (12%) [48], consuming a large portion of disposable incomes. In Hong Kong, 53% of the population lived in private housing, and 47% of the total population were tenants. The housing problem (including home ownership and access to affordable rental housing) was one of the most important issues. Although areas with a high populous grassroots score were scattered in the northern part of Hong Kong, most areas in northern Hong Kong were less developed, where decentralization of the population from the more crowded central parts of Hong Kong can be considered, through urban development programs.

#### 4.1.5. Principal Component 5: Young-Age

PC5 mainly focuses on aging vulnerability. It is not surprising that the last component was related to internal migration, since the residential mobility propensity for older people is constrained [49]. In Hong Kong, one-third of households had at least one older member aged 65 and above [50], accounting for 15.6% of the whole Hong Kong population in 2016. Low fertility rates and high life expectancy suggests that population ageing in Hong Kong will continue. Since population ageing puts pressure on medical and health care services, including changing the prevalence of diseases and raising expenses for medical care, health education activities and disease prevention measures should be enhanced, especially for areas with a low young-age score.

### 4.2. Concepts of the Five Principal Components

Despite different conceptual frameworks for defining and assessing vulnerability, two common factors of vulnerability have been identified in risk reduction or risk management studies [51,52,53,54], fragility and lack of coping capacity [55]. The former is about the capacity to survive or sustain under the impacts of a hazard, including the physical condition of the person and the security of the living environment, while the latter is about the capacity to access resources to react or respond to harmful impacts, including the availability of appropriate information, social support and income. PC5 and PC4 align with the factor of fragility as they represent something “inherent” in the geographical area [21], which operates before and after the occurrence of a hazard. They could only be modified at the macro governmental level through legislation, public private partnership and so on. PCs 1 to 3 correspond to the lack of coping capacities factor, which could be altered at the micro household or individual level via information seeking, social network strengthening and self-initiated educational and financial programs. In addition, by definition, the principal components were derived in decreasing order of variation, where PC 1 had the highest variability, supporting the argument that PCs 1 to 3 were potentially easier to be changed than PCs 4 and 5. Although both PCs 3 and 4 were income- or wealth-related, the effect of PC3 was transient, while PC4 was long-lasting.

### 4.3. Five Clusters 

#### 4.3.1. Cluster 1: Disadvantaged Inner-City Areas

Geographical areas of Cluster 1 were mainly located on the west and central northern coast of Hong Kong Island and northwestern Kowloon, with aging populations, high building density and limited access to open spaces. On the one hand, Cluster 1 included the traditional inner-city poor areas where the situation was exacerbated by aging. Since older people either lack employment income or engage in low-skill, low-income occupations, they face a higher risk of poverty. Targeted cash policy would benefit them the most. On the other hand, Cluster 1 also contains areas with low family resilience. The limited assistance from family members forces older people to rely on public social services which become of increasing importance to them.

#### 4.3.2. Cluster 2: Remote Rural Areas

Like those in Cluster 1, Cluster 2 areas lacked family resilience, but they included younger communities. Residential areas here were not as modern as the urban areas and were environmentally attractive, located in the periphery of the city. Nevertheless, some public infrastructure development projects were taking place to build an accessible open space and stimulate the growth of the adjacent areas.

#### 4.3.3. Cluster 3: Affluent Areas

In these areas, people were more likely to be wealthy and educated. However, it should be recognized that there were some vulnerable groups (i.e., foreign domestic helpers), associated with fewer economic resources and ethnic minority identity, which calls for sophisticated health services planning and delivery.

#### 4.3.4. Cluster 4: Suburban Areas

To tackle population growth and improve living environment through decentralizing from the over-crowded urban areas, the Hong Kong government has developed 12 new towns since the 1970s to accommodate public and private housing, infrastructure and facilities, external transport links to other areas, and job opportunities. The development of the 12 new towns was implemented in three phases. The first phase was initiated in the early 1970s, while the second and third phases commenced in the late 1970s and 1980s, respectively.

In this study, all the 12 new towns and a few adjacent TPUs, combined with some traditional urban areas fell into Cluster 4. It is worth noting that Tin Shui Wai, built on land reclaimed and with a wetland park, and North Lantau, part of the projects associated with the Hong Kong International Airport development, were identified as two outliers of the cluster. Both were developed in the third phase and with a shorter development time. Since these areas have high populous grassroots scores and low young-age scores, which are correlated with high mortality rate, special attention should be paid to them.

#### 4.3.5. Cluster 5: Developing Areas

These areas are in the process of urbanization, and still preserve some agricultural character. Inhabitants were relatively young but with low socioeconomic status. For certain historical reasons, approximately 40% of the total landmass in Hong Kong is protected by policy to remain undeveloped [56], and the government is undertaking planning and studies to review the feasibility for future development to attract more people to settle in these areas.

### 4.4. Limitations

This study has several limitations. Firstly, there is currently a data gap for individual level data; all variables were assessed at the TPU level. Thus, there is potential for an ecological fallacy (i.e., measurements at aggregate levels may not be representative at individual level), to arise. Secondly, mathematical methods are not designed with specific consideration of pragmatic implications. Although using the first few principal components to provide a summary of the original variables might prove useful, it can be difficult to reach a consensus in interpreting the meaning of and labeling each derived component, especially for the first principal component (PC1). This is because although the scores of the PC1 maximally discriminate all the observations, the meaning of PC1 may be quite obscure and might not be the most interesting for a study since it often presents merely a general profile of the observations, while PC2 and the subsequent components usually offer different measures on some specific aspects. Thirdly, the selected variables in the principal components analysis were subject to data availability, and the principal components generated were based on the understandings and experience in the field and the references considered. However, the socioeconomic data in this study showed a distinct pattern in analysis, having high correlations within a small set of socioeconomic variables and no or little correlation with others, enhancing the interpretability of the results. Fourthly, since data collection via the population census in different countries/areas may serve some locally specific purposes, the marked component structure in this study (e.g., the housing-related component) may not be important elsewhere, or the degree of importance of some principal components may appear in a reverse order in other places. Finally, the results presented were based on the 2016 population by-census in Hong Kong; the reported socioeconomic vulnerability and clustering were subject to change when new policies and development projects were conducted afterwards. The results should therefore be interpreted with caution and future studies are needed to capture the most up-to-date situation.

## 5. Conclusions

This paper shows how socioeconomic-vulnerability-related data may be categorized into five principal components, including indigenous degree, family resilience, individual productivity, populous grassroots, and young-age. Socioeconomic vulnerability is a concept that could be used to help identify susceptible areas, and even identify susceptible groups in affluent areas. Areas with a high populous grassroots score and a low young-age score should be paid more attention since they were correlated with a higher mortality rate.

## 6. Implication

Vulnerability reduction in populations is an important means of risk management. This study provides a parsimonious method of organizing a large, complex set of multivariate spatial socioeconomic data and identifies some health vulnerable geographical areas or subgroups in Hong Kong worth investigating. Following the clustering in this study, the government might consider implementing a similar sustainable urban development policy that has been implemented successful in one area to another area of the same cluster, based on the similarity of their socioeconomic (including urban planning) characteristics as grouped into the five components. Public health and urban planning policies, as well as risk communication programs, might then be tailored for the different areas as represented by the different clusters. However, this quantitative vulnerability assessment approach needs to be complemented with qualitative approaches to enhance full interpretability, and to capture the various tangible and intangible aspects of vulnerability.

Since public health policies and measures have a critical role to play in reducing and managing the negative health impact of emergencies or hazards, various global disaster risk reduction frameworks call for a stronger role of local governments and stakeholders, in particular at the non-emergency stage [57]. Systematic analysis of health-related risk is a prerequisite of risk reduction. The proposed vulnerability assessment scheme, as one of the applications of the Health-EDRM framework, can help assessment of the spatial distribution of socioeconomic vulnerability (i.e., drivers of vulnerability and health risk) in an urban city. Taking into account sustainable development and urban development it can, hopefully, contribute to enhancing the understanding of drivers of health risk in multiple dimensions, providing practical guidance to decision-makers and practitioners to reduce health risk, empowering the vulnerable and building health resilience, and achieving synergies among the Sendai Framework, the 2030 Agenda for Sustainable Development, and the New Urban Agenda.

## Figures and Tables

**Table 1:** Key indicators of socioeconomic vulnerability.

Contributor of Vulnerability	Concept	Indicator	Relevant Literature
Demography	Demography	1. total population of the sub-region	Donner & Rodríguez [19]
	Gender	2. sex ratio (ratio of males to females)	Chappell, Dujela, & Smith [11]; Sharma, Chakrabarti & Grover [12]
	Age	3. % of vulnerable age (age <15 or ≥65)	Chan et al. [17]; Wisner et al. [3]; Flagg et al. [9]; Welle and Birkmann [20]
		4. median age	
	Ethnicity	5. % of Chinese ethnicity	Wisner et al. [3]; Cutter, Boruff, & Shirley [18]; Enarson & Fordham [25]
	Language	6. % of usual spoken language as Cantonese	Wisner et al. [3]; Cutter, Boruff, & Shirley [18]
		7. % of able to read Chinese	
		8. % of able to read English	
		9. % of able to write Chinese	
		10. % of able to write English	
	Education	11. % of primary education attainment or below	Adger [2]; Wong et al. [22]
		12. % of studying inside the sub-region	
Poverty	Employment	13. % of employee	Phillimore, Beattie, & Townsend [13]; Wong et al. [22]
		14. labor force participation rate	
		15. % of working inside the sub-region	
	Occupation	16. % of white collar	Enarson & Fordham [25]
	Income	17. median individual income	Adger [2]; Hewitt [5]; Fothergill & Peek [26]; Welle and Birkmann [20]
		18. median household income	
Resource dependency	Household size	19. % of one-person household	Strachan [8]; Flagg et al. [9]; Emery et al. [10]; Wong et al. [22]
		20. average household size	
	Marital status	21. % of married	Wong et al. [22]
	Migration	22. % of migrated internally in Hong Kong	Adger [2]; Donner & Rodríguez [19]
Inequality	Housing	23. % of government housing	Phillimore, Beattie, & Townsend [13]; Moore [14]; Grander [15]; Tach & Emory [16]
		24. % of tenants	
		25. median household rent	
		26. median rent to income ratio	
		27. median floor area of accommodation	

## Data Availability

The data are not publicly available due to restrictions by the Census and Statistics Department of the Hong Kong SAR Government.
